# Supplementary material for: The effect of genetic variation on promoter usage and enhancer activity
Source: Nat Commun. 2017 Nov 7;8:1358. doi: 10.1038/s41467-017-01467-7 (PMC5677018; doi:10.1038/s41467-017-01467-7)
Supplement: Supplementary file 2 — Supplementary Information [file 41467_2017_1467_MOESM2_ESM.pdf]

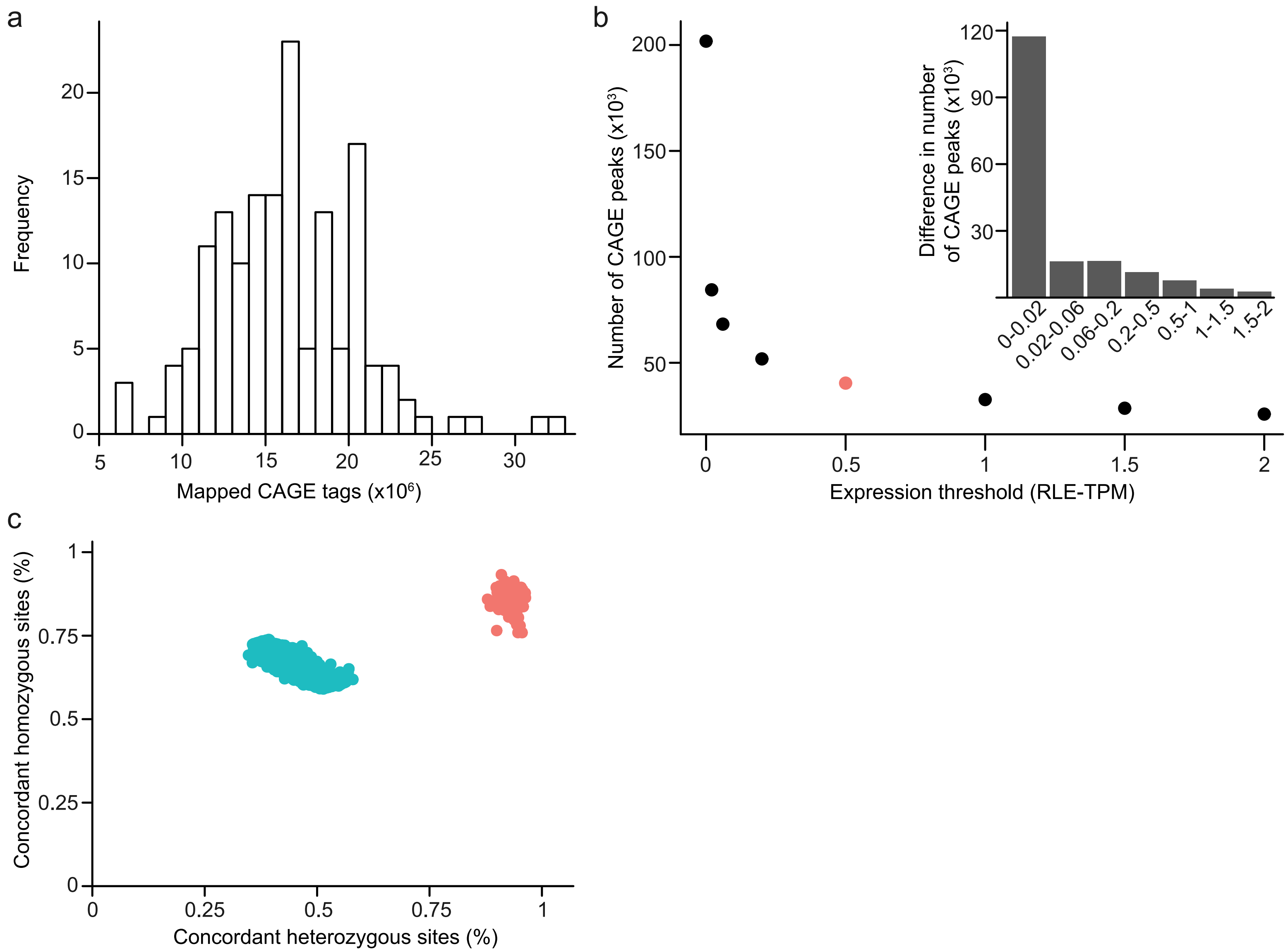

Supplementary Figure 1: Sequencing statistics and quality control. (**a**) Sequencing depth is shown as a histogram of single mapped CAGE tags (MapQ>20) for 154 individuals. (**b**) Distribution of CAGE peaks (including X and Y chromosomes) passing different expression thresholds. The 0.5 RLE-TPM threshold used in the study is highlighted in red. The bar-plot shows the number of CAGE peaks lost between consecutive thresholds. (**c**) *MBV* output (Methods) allelic consistency for heterozygous (x-axis) and homozygous (y-axis) variants, between genotype and CAGE tag sequences are plotted. Red dots indicate proper identity match of sequencing with expected genotypes. As control, we show consistency levels for non-matching samples (blue dots).

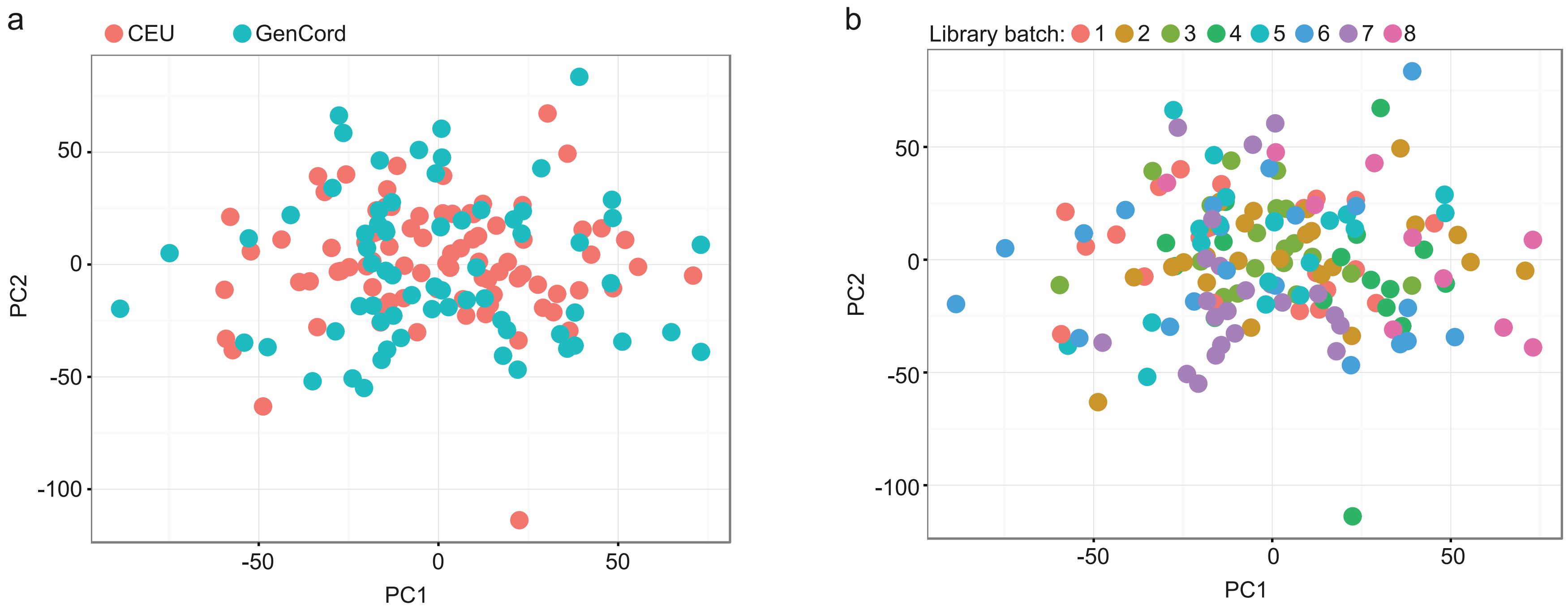

Supplementary Figure 2: Data stratification. Projection onto the first two principal components of the normalized data, residualized for the first 3 PCs derived from genotype data and the first 20 PCs of the promoter expression values, labelled for (**a**) the two cohorts used in the study and (**b**) for batches of library preparations.

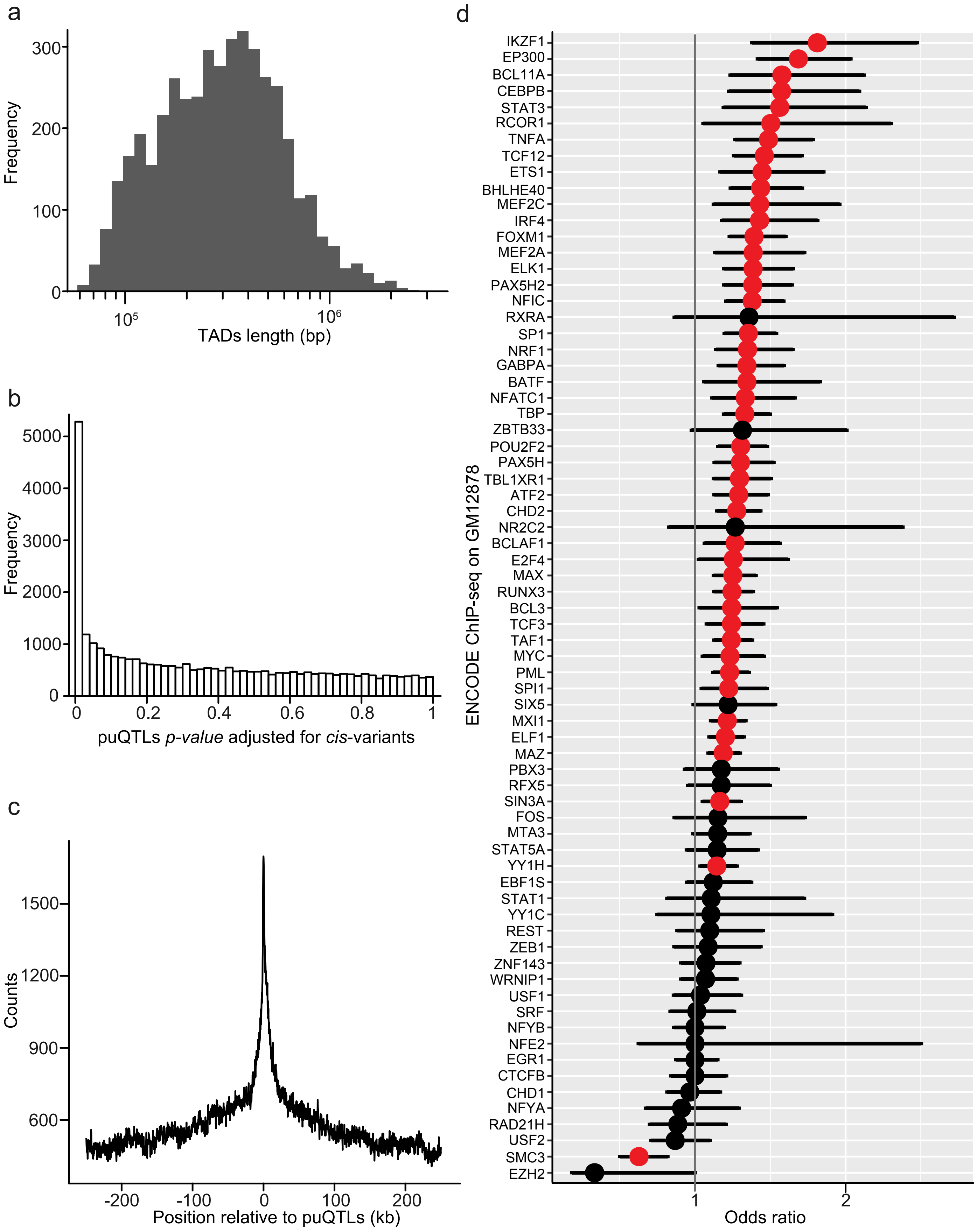

Supplementary Figure 3: puQTLs mapping and enrichment analyses. (**a**) Distribution of TADs length (**b**) Distribution of *p-values* adjusted for number of tested *cis*-variants associations with promoters. (**c**) Total counts of DNase I hypersensitivity signal (GM12878 cells, ENCODE data) for the 500 kb region flanking puQTLs. (**d**) Enrichment of transcription factor ChIP-seq signal (GM12878 cells, ENCODE data) at puQTL sites. Red dots indicate values passing the significance threshold of 5% FDR and bars show 95% confidence intervals.

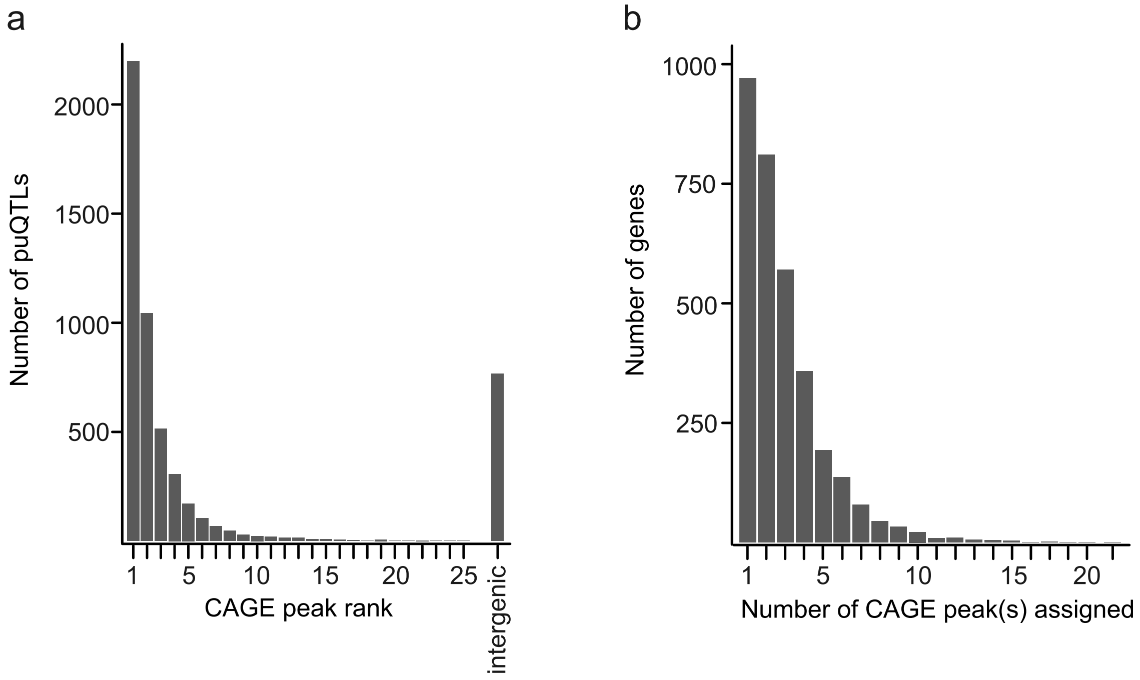

Supplementary Figure 4: FANTOM CAGE peaks annotation. (**a**) Distribution of puQTLs for the CAGE peak rank and (**b**) number of CAGE peaks per gene.

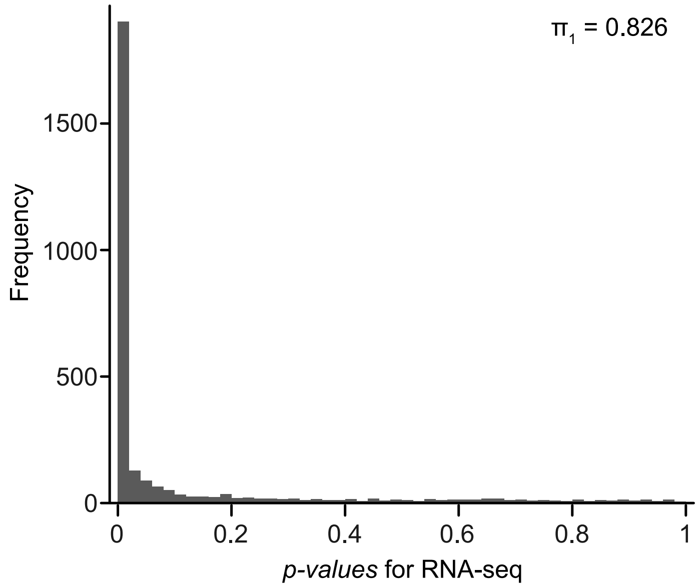

Supplementary Figure 5: π_1 statistic for puQTLs replicating eQTLs calling for mRNA levels measured from RNA-seq data.

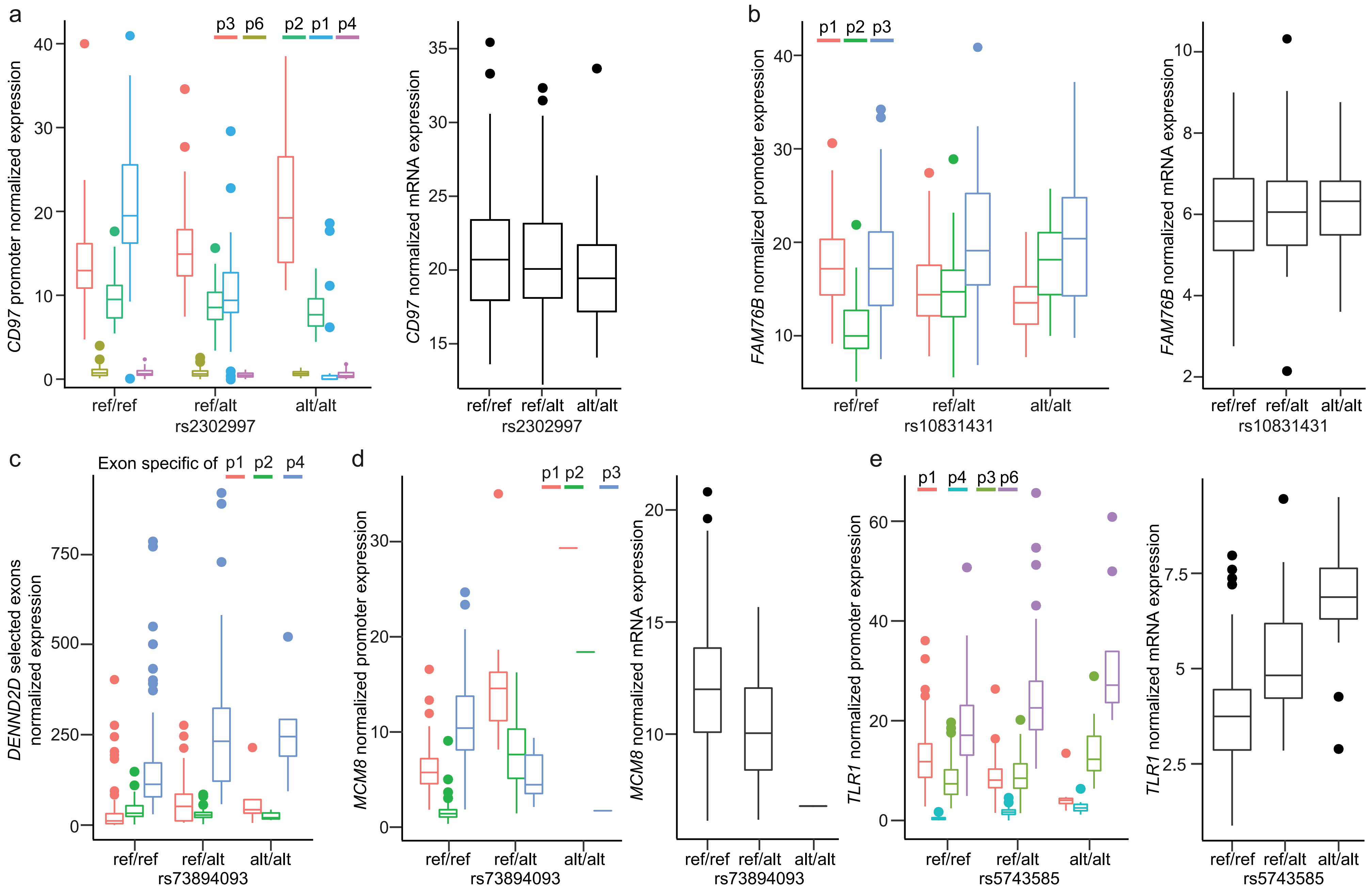

Supplementary Figure 6: Normalized expression for promoters (colored box plots) and mRNA levels (black box plots) relative to puQTL genotypes are plotted for *CD97* (**a**) and *FAM76B* (**b**) both belonging to the puQTLs associated gene group-3 without effect on mRNA levels. (**c**) Quantification of exons specific to different *DENND2D* isoforms are plotted for the different puQTL genotypes. Normalized expression for promoters and mRNA levels relative to puQTLs genotypes are plotted for *MCM8* (**d**) and *TLR1* (**e**) both belonging to the puQTLs associated gene group-3, with observed eQTL effect.

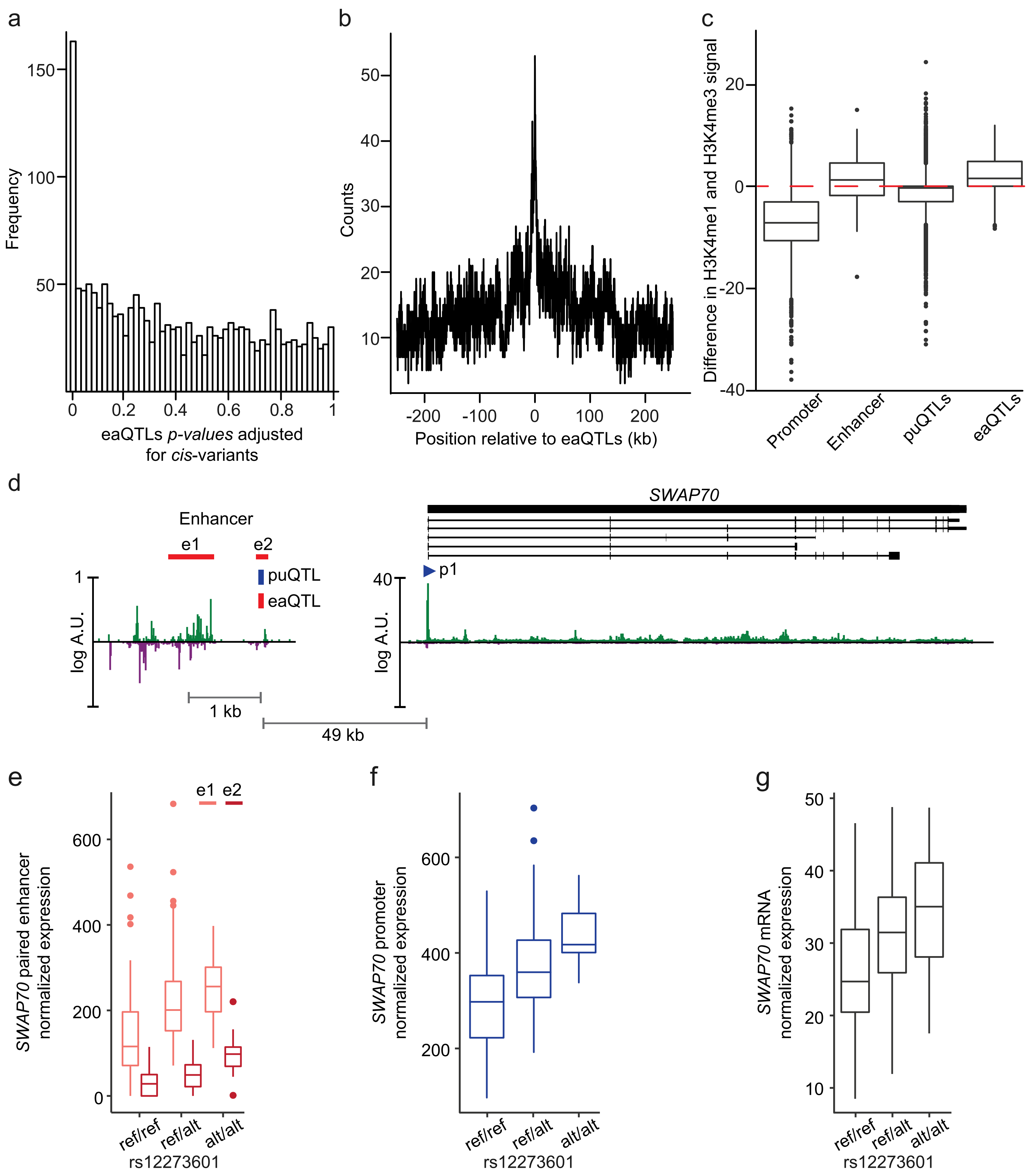

Supplementary Figure 7: eaQTLs mapping and integration with puQTLs. (**a**) Distribution of *p-values* adjusted for number of tested *cis*-variants associated with enhancers. (**b**) Total counts of DNase I hypersensitivity signals (GM12878 cells, ENCODE data) for the 500 kb region flanking eaQTLs. (**c**) Difference in raw H3K4me1 and H3K4me3 ChIP-seq signals for CAGE-peaks associated with puQTLs (promoters) and eaQTLs (enhancers). (**d**) CAGE signal at the *SWAP70* locus and associated enhancer region. The variant (rs12273601) mapped as puQTL for *SWAP70*-associated CAGE peak (p1) and eaQTL for paired enhancers (e1, e2) is shown. Normalized enhancer (**e**), promoter (**f**) and mRNA (**g**) expression relative to each genotype group are plotted for the entire population (n=154).

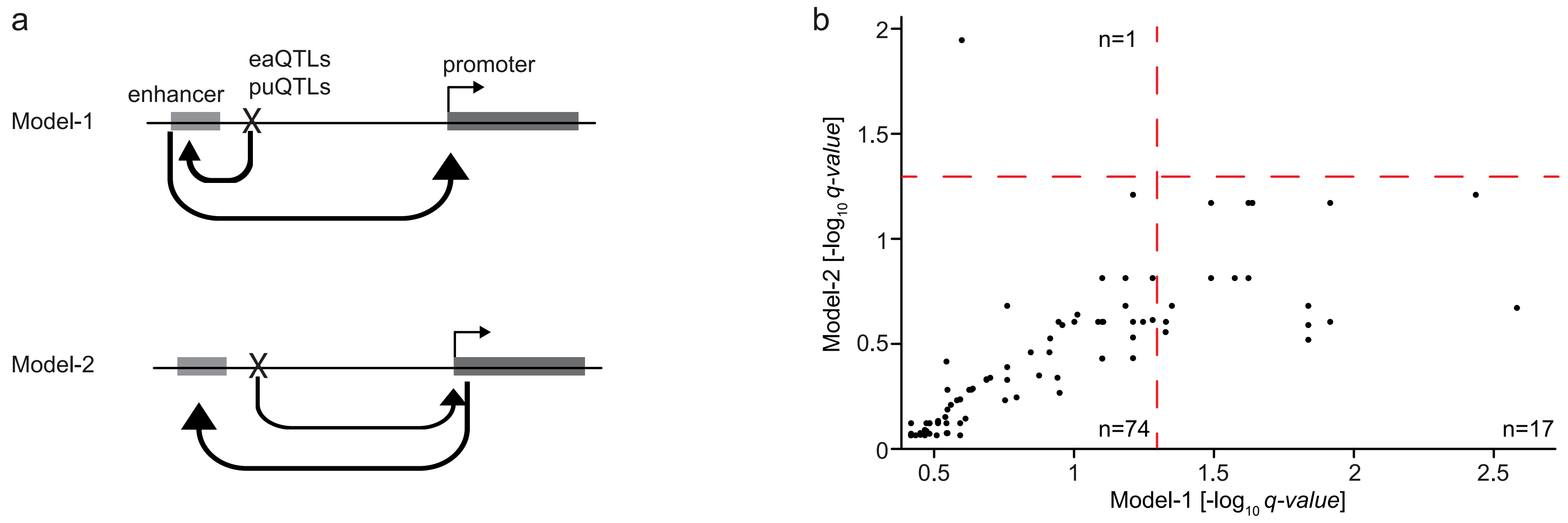

Supplementary Figure 8: assessing causal mediation of eaQTLs for two model networks (**a**) considering either the enhancer activity as mediator for promoter expression (Model-1) or reciprocally (Model-2). (**b**) Distribution of Causal inference testing associated *p-values* for the model networks are plotted for the 158 triplets composed of eaQTLs being also puQTLs, associated enhancers and paired promoters. Significance threshold of *q-values*<0.05 are shown (dashed lines). Number of tested trios in each significance group are indicated.

Supplementary Table 1: Samples sequencing information

Cell Repository	Sample_ID	Sex	Mapped_reads (MAPQ 20)	Reads redundancy
Coriell	GM06984	male	12640183	2.49
Coriell	GM06985	female	11969515	2.26
Coriell	GM06989	female	20785454	2.35
Coriell	GM07037	female	11086733	2.4
Coriell	GM07051	male	14214959	2.4
Coriell	GM07056	female	9624480	2.52
Coriell	GM07347	male	16019204	2.35
Coriell	GM07357	male	15366979	2.06
Coriell	GM10847	female	15738995	2.48
Coriell	GM10851	male	18737689	2.15
Coriell	GM11829	male	11997348	2.3
Coriell	GM11830	female	20129149	2.46
Coriell	GM11831	male	12089666	2.23
Coriell	GM11832	female	13325612	2.29
Coriell	GM11840	female	18607981	2.13
Coriell	GM11843	male	22446590	2.33
Coriell	GM11881	male	13457683	2.52
Coriell	GM11893	male	19222190	2.45
Coriell	GM11894	female	10845659	2.82
Coriell	GM11918	female	16428451	2.75
Coriell	GM11919	male	11620517	2.61
Coriell	GM11920	female	15256307	2.65
Coriell	GM11930	male	12900853	2.21
Coriell	GM11931	female	14685153	2.35
Coriell	GM11932	male	8830532	2.5
Coriell	GM11933	female	16240019	2.53
Coriell	GM11992	male	13437762	2.29
Coriell	GM11994	male	18510688	2.27
Coriell	GM11995	female	16192878	2.49
Coriell	GM12005	male	16652720	2.64
Coriell	GM12006	female	20833821	2.49
Coriell	GM12043	male	23134678	2.32
Coriell	GM12044	female	15669075	2.24
Coriell	GM12045	male	16296316	2.13
Coriell	GM12046	female	11436737	2.35
Coriell	GM12154	male	16587610	2.75
Coriell	GM12155	male	18328257	2.48
Coriell	GM12156	female	15833961	2.34
Coriell	GM12234	female	10459181	2.12
Coriell	GM12249	female	18191955	2.26
Coriell	GM12272	male	18039600	2.26
Coriell	GM12275	female	23622720	2.83
Coriell	GM12282	male	16212900	2.42
Coriell	GM12283	female	19698012	2.11
Coriell	GM12286	male	11719353	2.37
Coriell	GM12287	female	14583532	2.33
Coriell	GM12340	male	9413406	1.98
Coriell	GM12341	female	14618466	2.54
Coriell	GM12347	male	15614874	2.2
Coriell	GM12348	female	10523527	2.32
Coriell	GM12383	female	11837449	2.35
Coriell	GM12399	male	12531444	2.52

Coriell	GM12400	female	18129412	2.26
Coriell	GM12413	male	14593987	2.25
Coriell	GM12414	female	14688668	2.34
Coriell	GM12489	female	16032866	2.62
Coriell	GM12546	male	16380797	2.36
Coriell	GM12716	male	20138992	2.35
Coriell	GM12717	female	6800531	2.38
Coriell	GM12718	female	12489277	2.38
Coriell	GM12748	male	20231128	2.43
Coriell	GM12749	female	16052947	2.62
Coriell	GM12750	male	20581013	2.36
Coriell	GM12751	female	16631894	2.15
Coriell	GM12760	male	16089084	2.13
Coriell	GM12761	female	16596991	2.06
Coriell	GM12762	male	14281839	2.19
Coriell	GM12763	female	31132009	2.32
Coriell	GM12775	male	14567915	2.32
Coriell	GM12776	female	16336452	2.12
Coriell	GM12777	male	12561614	1.95
Coriell	GM12778	female	12540858	2.01
Coriell	GM12813	female	11092562	2.2
Coriell	GM12814	male	21212043	2.51
Coriell	GM12815	female	15694239	2.55
Coriell	GM12827	male	14480625	2.31
Coriell	GM12828	female	12947084	2.36
Coriell	GM12830	female	20600972	2.6
Coriell	GM12842	male	16326180	2.19
Coriell	GM12843	female	16237630	2.06
Coriell	GM12872	male	13987532	2.22
Coriell	GM12873	female	17346750	2.59
Coriell	GM12874	male	15848655	2.05
Coriell	GM12878	female	14473727	2.47
Coriell	GM12889	male	20776334	2.29
Coriell	GM12890	female	18365335	2.32
GenCord	UCB1001	female	11247126	2.05
GenCord	UCB1011	male	6813096	2.2
GenCord	UCB1012	male	13519148	2.36
GenCord	UCB1014	female	9561598	1.77
GenCord	UCB1016	female	17149833	2.37
GenCord	UCB1023	female	16187036	2.33
GenCord	UCB1025	male	16181650	2.53
GenCord	UCB1027	female	6816136	1.81
GenCord	UCB1029	male	12970208	2.05
GenCord	UCB1046	male	15058736	2.34
GenCord	UCB1048	male	26478690	2.3
GenCord	UCB1052	female	15574523	2.26
GenCord	UCB1053	female	14323582	2.3
GenCord	UCB1056	female	17783349	2.15
GenCord	UCB1058	female	20343911	2.41
GenCord	UCB1061	male	18420911	2.33
GenCord	UCB1064	male	18428398	2.4
GenCord	UCB1067	male	18870632	2.31
GenCord	UCB1069	female	17026777	2.09
GenCord	UCB1070	female	13599139	2.31
GenCord	UCB1071	female	20357006	2.61
GenCord	UCB1072	male	9735829	2.77
GenCord	UCB1073	male	14383787	2.7

GenCord	UCB1074	female	20649343	2.43
GenCord	UCB1075	male	18363285	2.17
GenCord	UCB1076	female	20469043	2.69
GenCord	UCB1077	male	22178879	2.72
GenCord	UCB1079	male	16944460	2
GenCord	UCB1080	male	16008585	2.21
GenCord	UCB1083	female	20729535	2.18
GenCord	UCB1086	male	18305441	2.3
GenCord	UCB1087	male	20933257	2.14
GenCord	UCB1089	male	21579957	2.33
GenCord	UCB1091	female	18696951	2.36
GenCord	UCB1092	female	13986946	2.17
GenCord	UCB1094	female	19336987	2.23
GenCord	UCB1096	male	27990927	2.7
GenCord	UCB1098	male	20290931	2.33
GenCord	UCB1099	female	16722729	2.28
GenCord	UCB1100	male	14315030	2.69
GenCord	UCB1101	male	32925680	2.35
GenCord	UCB1102	female	13679271	2.61
GenCord	UCB1103	male	15781322	2.78
GenCord	UCB1105	male	15540940	2.37
GenCord	UCB1107	male	19546188	2.26
GenCord	UCB1108	male	21124930	2.25
GenCord	UCB1112	male	15085524	2.61
GenCord	UCB1113	male	20592220	2.23
GenCord	UCB1117	female	19965337	2.75
GenCord	UCB1119	female	12269023	2.29
GenCord	UCB1120	male	22902656	2.61
GenCord	UCB1129	male	22146555	2.8
GenCord	UCB1130	male	11452870	2.37
GenCord	UCB1131	female	11884848	1.79
GenCord	UCB1136	female	24912880	2.73
GenCord	UCB1139	female	10515970	2.04
GenCord	UCB1148	female	10805484	2.49
GenCord	UCB1196	female	12034373	2.74
GenCord	UCB1199	female	12953368	2.02
GenCord	UCB1200	male	12520479	2.48
GenCord	UCB1203	male	13606302	1.78
GenCord	UCB1206	male	21348821	2.59
GenCord	UCB1207	female	20464177	2.28
GenCord	UCB1208	male	13498234	2.22
GenCord	UCB1210	male	16064381	2.1
GenCord	UCB1212	male	15870193	2.18
GenCord	UCB1216	female	17609259	2.77
GenCord	UCB1218	male	14569047	2.1

Supplementary Table 2: puQTLs linked with GWAS hits

puQTLs (hg19, chromosome_start)	puQTLs associated promoter (hg19; chr_start_end_strand)	Promoter associated gene or regulatory element	GWAS hit (hg19, chromosome_start)	GWAS hit (rs ID)	RTC value	GWAS catalog reported trait(s) (reference PMID)
chr8_11338383	chr8_11324375_11324387_-	*FAM167A* (ENSG00000154319.10)	chr8_11339965	rs2736332	1	systemic lupus erythematosus (PMID:27399966;26502338)
chr8_11339965	chr8_11324273_11324289_-	*FAM167A* (ENSG00000154319.10)	chr8_11339965	rs2736332	1	systemic lupus erythematosus (PMID:27399966;26502338)
chr8_11351019	chr8_11313812_11313825_-	intronic enhancer element	chr8_11339965	rs2736332	1	systemic lupus erythematosus (PMID:27399966;26502338)
chr8_11351912	chr8_11313523_11313532_-	intronic (*FAM167A*)	chr8_11339965	rs2736332	1	systemic lupus erythematosus (PMID:27399966;26502338)
chr8_11352541	chr8_11313428_11313437_-	intronic (*FAM167A*)	chr8_11352541	rs2618476	1	systemic lupus erythematosus (PMID:19165918)
chr8_11352541	chr8_11313428_11313437_-	intronic (*FAM167A*)	chr8_11339965	rs2736332	0.923	systemic lupus erythematosus (PMID:27399966;26502338)
chr8_11395232	chr8_11351905_11351925_+	*BLK* (ENSG00000136573.8)	chr8_11395232	rs1478897	1	systemic lupus erythematosus (PMID:26316170)
chr8_11403769	chr8_11403533_11403544_+	*BLK* (ENSG00000136573.8)	chr8_11339965	rs2736332	1	systemic lupus erythematosus (PMID:27399966;26502338)
chr8_11453671	chr8_11665932_11665943_+	*FDFT1* (ENSG00000079459.8)	chr8_11352541	rs2618476	0.923	systemic lupus erythematosus (PMID:19165918)
chr1_67731368	chr1_67519812_67519854_-	*SLC35D1* (ENSG00000116704.6)	chr1_67731368	rs7547569	1	inflammatory bowel disease (PMID:26192919)
chr13_44453480	chr13_44453833_44453912_-	*CCDC122* (ENSG00000151773.8)	chr13_44457925	rs3764147	1	inflammatory bowel disease (PMID:26192919); Crohn's disease (PMID:23128233;21102463;18587394); leprosy (PMID:20018961)
chr13_44457925	chr13_44453688_44453795_+	*LACC1* (ENSG00000179630.6)	chr13_44457925	rs3764147	1	inflammatory bowel disease (PMID:26192919); Crohn's disease (PMID:23128233;21102463;18587394); leprosy (PMID:20018961)
chr16_28837515	chr16_28857630_28857675_-	*TUFM* (ENSG00000178952.4)	chr16_28837515	rs8049439	1	inflammatory bowel disease (PMID:19915574); educational attainment (PMID:27225129;25201988;23722424)
chr19_10512400	chr19_10515518_10515566_+	intronic enhancer element	chr19_10512911	rs11879191	1	inflammatory bowel disease (PMID:23128233); Crohn's disease (PMID:26192919)
chr19_10512911	chr19_10515604_10515629_+	intronic enhancer element	chr19_10512911	rs11879191	1	inflammatory bowel disease (PMID:23128233); Crohn's disease (PMID:26192919)
chr9_4985879	chr9_4985228_4985245_+	*JAK2* (ENSG00000096968.8)	chr9_4985879	rs2274471	1	Crohn's disease (PMID:23266558)
chr19_40170053	chr19_40170009_40170016_+	*LGALS17A* (ENSG00000226025.5)	chr19_40170053	rs8103033	1	obesity-related traits (PMID:23251661)
chr22_42326181	chr22_42342702_42342716_-	*CENPM* (ENSG00000100162.10)	chr22_42336172	rs5758511	1	birth weight (PMID:23202124)
chr22_42336172	chr22_42336209_42336228_-	*CENPM* (ENSG00000100162.10)	chr22_42336172	rs5758511	1	birth weight (PMID:23202124)
chr3_16413520	chr3_16524357_16524377_-	*RFTN1* (ENSG00000131378.9)	chr3_16417555	rs493733	1	urate levels, BMI interaction (PMID:25811787)
chr3_16417555	chr3_16553570_16553583_+	antisense transcript of *RFTN1*	chr3_16417555	rs493733	1	urate levels, BMI interaction (PMID:25811787)
chr3_52648265	chr3_52719912_52719995_-	*PBRM1* (ENSG00000163939.14)	chr3_52649748	rs13083798	1	waist-hip ratio adjusted for body mass index (PMID:25673412)
chr3_52649748	chr3_52720016_52720057_+	*GNL3* (ENSG00000163938.12)	chr3_52649748	rs13083798	1	waist-hip ratio adjusted for body mass index (PMID:25673412)
chr1_11862778	chr1_11866270_11866314_+	*CLCN6* (ENSG00000011021.17)	chr1_11862778	rs17367504	1	blood presure (PMID:21909110); systolic blood pressure (PMID:19430483)
chr1_89467242	chr1_89458607_89458627_-	*CCBL2* (ENSG00000213516.5)	chr1_89474818	rs17433780	1	carotid artery intima media thickness (PMID:26343869)
chr1_89474818	chr1_89458651_89458685_-	*CCBL2* (ENSG00000213516.5)	chr1_89474818	rs17433780	1	carotid artery intima media thickness (PMID:26343869)
chr1_89618472	chr1_89458415_89458463_-	*CCBL2* (ENSG00000213516.5)	chr1_89474818	rs17433780	1	carotid artery intima media thickness (PMID:26343869)
chr2_203808532	chr2_203736475_203736503_-	*ICA1L* (ENSG00000163596.12)	chr2_203808532	rs140244541	1	low density lipoprotein cholesterol measurement (PMID:25961943)
chr2_85788175	chr2_85788605_85788629_-	*GGCX* (ENSG00000115486.7)	chr2_85788175	rs7568458	1	coronary artery disease (PMID:26343387)
chr15_78817929	chr15_78832757_78832806_+	*PSMA4* (ENSG00000041357.11)	chr15_78817929	rs8042849	1	forced expiratory volume post bronchodilator (PMID:26634245)
chr15_78817929	chr15_78832757_78832806_+	*PSMA4* (ENSG00000041357.11)	chr15_78821016	rs12441354	1	forced expiratory volume post bronchodilator (PMID:26634245)
chr15_78821016	chr15_78832993_78833010_-	antisense transcript of *PSMA4*	chr15_78821016	rs12441354	1	forced expiratory volume post bronchodilator (PMID:26634245)
chr15_78857812	chr15_78857836_78857847_+	*CHRNA5* (ENSG00000169684.9)	chr15_78817929	rs8042849	1	forced expiratory volume post bronchodilator (PMID:26634245)
chr15_78858400	chr15_78857870_78857897_+	*CHRNA5* (ENSG00000169684.9)	chr15_78858400	rs684513	1	forced expiratory volume post bronchodilator (PMID:26634245)
chr1_155194980	chr1_155197244_155197268_-	*GBAP1* (ENSG00000160766.10)	chr1_155194980	rs2049805	1	renal function related traits (PMID:22797727)
chr11_44087989	chr11_44088027_44088086_+	*ACCS* (ENSG00000110455.9)	chr11_44087989	rs2074038	1	IGA glomerulonephritis (PMID:26028593)
chr20_23612737	chr20_23618615_23618627_-	*CST3* (ENSG00000101439.4)	chr20_23612737	rs911119	1	chronic kidney disease (PMID:20383146)
chr11_61595564	chr11_61595533_61595544_+	*FADS2* (ENSG00000134824.9)	chr11_61595564	rs968567	1	rheumatoid arthritis (PMID:24390342); glycerophospholipid levels (PMID:26068415); blood metabolite levels (PMID:24816252)
chr22_39747671	chr22_39745939_39746012_+	*SYNGR1* (ENSG00000100321.10)	chr22_39747671	rs909685	1	rheumatoid arthritis (PMID:24390342)
chr13_76120727	chr13_76123613_76123659_+	*UCHL3* (ENSG00000118939.13)	chr13_76136648	rs9543976	1	diabetic retinopathy (PMID:23562823)
chr13_76136648	chr13_76123883_76123949_+	*UCHL3* (ENSG00000118939.13)	chr13_76136648	rs9543976	1	diabetic retinopathy (PMID:23562823)
chr12_56401085	chr12_56435881_56435936_+	*RPS26* (ENSG00000197728.5)	chr12_56401085	rs10876864	1	vitiligo (PMID:22951725)
chr12_56466654	chr12_56122977_56123016_+	*CD63* (ENSG00000135404.7)	chr12_56401085	rs10876864	1	vitiligo (PMID:22951725)
chr1_150826831	chr1_150947192_150947209_-	*CERS2* (ENSG00000143418.15)	chr1_150860471	rs7412746	1	melanoma (PMID:21983785)
chr1_150860471	chr1_150738211_150738260_-	*CTSS* (ENSG00000163131.6)	chr1_150860471	rs7412746	1	melanoma (PMID:21983785)
chr10_131265545	chr10_131265443_131265495_+	*MGMT* (ENSG00000170430.9)	chr10_131265545	rs16906252	1	*MGMT* methylation in smokers (PMID:26183928)
chr14_24609147	chr14_24610694_24610724_-	*FAM158A* (ENSG00000100908.9)	chr14_24609147	rs8010715	1	serum IgG glycosylation measurement (PMID:23382691)
chr16_792190	chr16_777097_777112_+	*HAGHL* (ENSG00000103253.13)	chr16_792190	rs11648796	1	body height (PMID:25429064;20881960)
chr19_2214057	chr19_2236246_2236286_-	*PLEKHJ1* (ENSG00000104886.5)	chr19_2214057	rs2074552	1	plasma omega-6 polyunsatureated fatty avid levels (PMID:26584805)
chr2_208012509	chr2_208031064_208031075_+	antisense transcript of *KLF7*	chr2_208012509	rs2284932	1	lachrymal and salivary gland lesion in type 1 autoimmune pancreatitis (PMID:25985088)
chr5_125918148	chr5_125930877_125930919_-	*ALDH7A1* (ENSG00000164904.11)	chr5_125918148	rs13182402	1	osteoporosis (PMID:20072603)
chr6_147531081	chr6_147524975_147524988_+	*STXBP5* (ENSG00000164506.10)	chr6_147531081	rs2786189	1	bronchopulmonary dysplasia (PMID:23897914)
chr6_32604372	chr6_32610846_32610863_+	*HLA-DQA1* (ENSG00000196735.7)	chr6_32604372	rs9272346	1	asthma (PMID:23181788); type I diabetes mellitus (PMID:18978792;17554300)

Supplementary Table 3: Mapped reads of GEUVADIS RNA-seq samples

Sample_ID	Mapped_reads
GM06984	2.39E+07
GM06985	4.27E+07
GM06986	4.51E+07
GM06989	2.66E+07
GM06994	4.74E+07
GM07037	3.54E+07
GM07048	3.24E+07
GM07051	3.47E+07
GM07056	3.24E+07
GM07347	4.28E+07
GM07357	3.32E+07
GM10847	4.05E+07
GM10851	3.53E+07
GM11829	2.71E+07
GM11830	3.25E+07
GM11831	5.35E+07
GM11832	4.62E+07
GM11840	3.46E+07
GM11843	3.80E+07
GM11881	3.85E+07
GM11892	2.76E+07
GM11893	5.39E+07
GM11894	4.59E+07
GM11918	3.12E+07
GM11920	4.16E+07
GM11930	2.68E+07
GM11931	3.29E+07
GM11992	2.99E+07
GM11994	5.85E+07
GM11995	3.41E+07
GM12004	2.52E+07
GM12005	3.97E+07
GM12006	4.38E+07
GM12043	4.15E+07
GM12044	4.12E+07
GM12045	2.01E+07
GM12058	4.07E+07
GM12144	4.06E+07
GM12154	3.59E+07
GM12155	3.19E+07
GM12156	4.02E+07
GM12234	4.44E+07
GM12249	3.55E+07
GM12272	2.31E+07
GM12273	3.41E+07
GM12275	3.83E+07
GM12282	2.02E+07
GM12283	3.22E+07
GM12286	3.87E+07
GM12287	1.87E+07
GM12340	3.94E+07
GM12341	3.86E+07
GM12342	3.45E+07
GM12347	3.85E+07
GM12348	1.92E+07
GM12383	3.21E+07
GM12399	1.53E+07
GM12400	3.30E+07
GM12413	4.99E+07
GM12489	3.81E+07
GM12546	2.54E+07
GM12716	9.19E+07
GM12717	3.55E+07
GM12718	4.39E+07
GM12749	2.64E+07
GM12750	3.63E+07
GM12751	4.90E+07
GM12760	1.63E+07
GM12761	4.18E+07
GM12762	2.39E+07
GM12763	4.29E+07
GM12775	5.73E+07
GM12776	4.17E+07
GM12777	3.52E+07
GM12778	3.20E+07
GM12812	2.68E+07
GM12813	3.48E+07
GM12814	5.71E+07
GM12815	3.97E+07
GM12827	1.98E+07
GM12829	5.36E+07
GM12830	2.45E+07

GM12842	4.41E+07
GM12843	2.99E+07
GM12872	4.30E+07
GM12873	3.92E+07
GM12874	3.62E+07
GM12889	4.37E+07
GM12890	3.08E+07
GM20502	3.22E+07
GM20503	2.88E+07
GM20504	3.53E+07
GM20505	3.40E+07
GM20506	2.91E+07
GM20507	3.47E+07
GM20508	3.73E+07
GM20509	7.60E+07
GM20510	1.74E+07
GM20512	3.46E+07
GM20513	3.88E+07
GM20514	2.99E+07
GM20515	4.81E+07
GM20516	4.05E+07
GM20517	2.71E+07
GM20518	3.14E+07
GM20519	4.15E+07
GM20520	1.82E+07
GM20521	3.04E+07
GM20524	4.40E+07
GM20525	2.97E+07
GM20527	5.29E+07
GM20528	4.14E+07
GM20529	4.01E+07
GM20530	4.74E+07
GM20531	3.97E+07
GM20532	2.36E+07
GM20534	4.54E+07
GM20535	3.30E+07
GM20536	2.99E+07
GM20538	2.06E+07
GM20539	3.77E+07
GM20540	2.47E+07
GM20541	4.03E+07
GM20542	2.36E+07
GM20543	3.60E+07
GM20544	3.70E+07
GM20581	2.93E+07
GM20582	4.46E+07
GM20585	2.64E+07
GM20586	4.29E+07
GM20588	4.03E+07
GM20589	3.57E+07
GM20752	4.75E+07
GM20754	3.55E+07
GM20756	3.77E+07
GM20757	3.81E+07
GM20758	5.59E+07
GM20759	2.30E+07
GM20760	2.36E+07
GM20761	3.61E+07
GM20765	5.50E+07
GM20766	3.26E+07
GM20768	3.68E+07
GM20769	2.80E+07
GM20770	2.01E+07
GM20771	4.00E+07
GM20772	3.82E+07
GM20773	3.33E+07
GM20774	2.40E+07
GM20778	1.25E+07
GM20783	4.27E+07
GM20785	3.46E+07
GM20786	4.53E+07
GM20787	4.34E+07
